# Tax1 binding protein 3 regulates osteogenic and adipogenic differentiation through inactivating Wnt/β‐catenin signalling

**DOI:** 10.1111/jcmm.17702

**Published:** 2023-03-09

**Authors:** Yi Wang, Ying Gan, Yuan Dong, Jie Zhou, Endong Zhu, Hairui Yuan, Xiaoxia Li, Baoli Wang

**Affiliations:** ^1^ NHC Key Lab of Hormones and Development, Tianjin Key Lab of Metabolic Diseases, Chu Hsien‐I Memorial Hospital & Institute of Endocrinology Tianjin Medical University Tianjin China; ^2^ College of Basic Medical Sciences Tianjin Medical University Tianjin China

**Keywords:** adipocyte, bone morphogenetic proteins, differentiation, osteoblast, Smads, Tax1bp3, β‐catenin

## Abstract

Tax1 binding protein 3 (Tax1bp3) is a PDZ domain‐containing protein that is overexpressed in cancer. Previous studies recognized Tax1bp3 as an inhibitor of β‐catenin. Till now it is not known whether Tax1bp3 regulates osteogenic and adipogenic differentiation of mesenchymal progenitor cells. In the current study, the data showed that Tax1bp3 was expressed in bone and was increased in the progenitor cells when induced toward osteoblast and adipocyte differentiation. The overexpression of Tax1bp3 in the progenitor cells inhibited osteogenic differentiation and conversely stimulated adipogenic differentiation, and the knockdown of Tax1bp3 affected the differentiation of the progenitor cells oppositely. Ex vivo experiments using the primary calvarial osteoblasts from osteoblast‐specific Tax1bp3 knock‐in mice also demonstrated the anti‐osteogenic and pro‐adipogenic function of Tax1bp3. Mechanistic investigations revealed that Tax1bp3 inhibited the activation of canonical Wnt/β‐catenin and bone morphogenetic proteins (BMPs)/Smads signalling pathways. Taken together, the current study has provided evidences demonstrating that Tax1bp3 inactivates Wnt/β‐catenin and BMPs/Smads signalling pathways and reciprocally regulates osteogenic and adipogenic differentiation from mesenchymal progenitor cells. The inactivation of Wnt/β‐catenin signalling may be involved in the reciprocal role of Tax1bp3.

## 1 INTRODUCTION

Osteoporosis is a common degenerative disease of bones, which leads to increased fragility and decreased strength of bones, making the bones prone to fracture.^1^ In bone, the major effector cells are osteoblasts, which are responsible for bone formation, and osteoclasts, which take charge of bone resorption.^2^ For the maintenance of healthy state of bone, it is of critical importance to maintain the balance between bone formation and resorption. Thus the in‐depth investigation and clarification of the molecular mechanism controlling osteoblast differentiation and bone formation will provide new insights for the prevention and treatment of osteoporosis.

Osteoblasts are derived from bone marrow stromal stem cells (BMSCs), which also give rise to adipocytes, myoblasts and chondrocytes.^3^ Under normal physiological conditions, BMSCs have similar potential to differentiate toward osteoblasts and adipocytes.^4^ A variety of transcription factors and signalling pathways, including runt‐related transcription factor‐2 (Runx2), osterix (OSX), canonical Wnt signalling pathway, and peroxisome proliferator activated receptor γ (PPARγ), reciprocally regulate osteogenic and adipogenic differentiation of BMSCs.^3^, ^4^, ^5^, ^6^, ^7^, ^8^

Tax1 binding protein 3 (Tax1bp3), also known as Tax interacting protein 1 (TIP1), is a 124 amino acid cytoplasmic protein that has been identified as the binding partner of Tax oncoprotein of human T‐lymphotropic virus type 1 (HTLV‐1).^9^ Tax1bp3 contains a 89‐amino‐acid PSD‐95/DlgA/ZO‐1 (PDZ) domain which is found in hundreds of proteins and has been recognized as an essential structural unit mediating protein interactions in intercellular contact, signal transduction and complex assembly of biological machineries. The single PDZ domain is the only structural and functional unit in Tax1bp3 that distinguishes the small protein from other PDZ proteins which more often contain multiple protein domains and function as scaffolds for protein complex assembly. Previous studies have revealed that Tax1bp3 is highly conserved across species^10^ and it is expressed in various cells and tissues.^10^, ^11^ The developmental role of Tax1bp3 has been documented in heart and brain.^10^ Besides, the high expression of Tax1bp3 was associated with adhesion, migration and metastasis of cancer cells, suggesting its function in tumorigenesis.^12^, ^13^, ^14^ Recently, it has been reported that Tax1bp3 can be used as an appropriate target for tumour immunotherapy and imaging of cancers, and the monoclonal antibody against Tax1bp3 can be used as a radio‐immunoconjugate moiety to specifically image and treat tumours.^15^

To date, it is not known if and how Tax1bp3 regulates the directional differentiation of mesenchymal stem cells. In the current study, we have for the first time investigated and uncovered the novel role of Tax1bp3 in regulating the osteogenic and adipogenic differentiation of mesenchymal progenitor cells.

## 2 METHODS

### 2.1 Cell cultures

Mouse BMSCs were isolated from the femurs and tibias by using the standard method of whole bone marrow direct adherence.^16^ Briefly, the tibias and femurs of 6‐week‐old mice were excised and the marrow cells were flushed using DMEM, and then grown on a 10 cm dish in a‐MEM containing 10% foetal bovine serum (FBS). Stromal line ST2, mesenchymal line C3H10T1/2 and preosteoblastic line MC3T3‐E1 cells were cultured in a‐MEM containing 10% FBS. At passage 3–6, the cells were induced to differentiate with osteogenic reagent when reaching 80% confluence, or induced to differentiate with adipogenic reagent when reaching 100% confluence, in a‐MEM containing 10% foetal bovine serum (FBS) following the previously published protocol.^16^

### 2.2 Generation of conditional Tax1bp3 knock‐in mice

A Cre‐responsive Tax1bp3 conditional knock‐in mouse was made in GemPharmatech. The mouse was generated by incorporating a Tax1bp3 expression cassette into the H11 locus. In brief, the expression cassette of the donor targeting construct contains a ubiquitous CAG promoter, which is responsible for the efficient expression of the transgene. A loxP‐stop‐loxP (LSL) cassette was inserted downstream of the promoter, which renders the transgene expression controlled by Cre recombinase. Next to the LSL cassette comes the Tax1bp3 coding sequence linked to a mCherry sequence via an IRES site to facilitate visualization of Tax1bp3‐expressing cells. The donor targeting construct was microinjected in the pronuclei of the fertilized eggs along with sgRNA and Cas9 mRNA. The LSL‐Tax1bp3 mice were crossed with Col1a1‐Cre transgenic mice to generate the knock‐in heterozygote line Col1a1‐Cre; LSL‐Tax1bp3/Wt. The genotypes of the mice were confirmed by genotyping PCR using a Col1a1‐cre specific primer and an LSL‐Tax1bp3 specific primer (Table S1).

The animal experiments were carried out following the National Standard of Animal Care and Use Procedures, and was approved by the Animal Ethics Committee of Tianjin Medical University Chu Hsien‐I Memorial Hospital.

### 2.3 Quantitative RT‐PCR

Total RNA was isolated from cultured cells using a kit (Omega Bio‐Tek) or from tissues using the RNA‐easy isolation reagent (Vazyme). RNA was reverse transcribed into cDNA using RevertAid first‐strand cDNA synthesis kit (Thermo Fisher). Quantitative PCR amplifications were performed in 20 μL reactions using SYBR Green PCR Master Mix (Abclonal). Primer sequences are listed in Table S1. β‐actin was used as a reference gene for normalization and the relative expression levels of the target genes were calculated using the 2^−ΔΔCt^ method.^17^

### 2.4 Plasmids, siRNAs and transfections

The full‐length CDS sequence of Tax1bp3 was PCR‐amplified, and the product was cloned into pcDNA3.1 vector using the pEASY®‐Basic Seamless Cloning and Assembly Kit (Transgen Biotech). To perform gain‐of‐function studies, the progenitor cells at 70% confluence were transfected for 4 h with Tax1bp3 expression plasmid or the empty plasmid by using JetPRIME transfection reagent (Polyplus). For the loss‐of‐function experiments, the progenitor cells at 50% confluence were transfected for 20 h with 20 nM Tax1bp3 siRNAs or negative control siRNA (Genepharma) using lipofectamine RNAi‐Max (Life technologies). After the transfections, the cells at appropriate confluence were induced to differentiate toward osteoblasts or adipocytes. The sequences of the siRNAs are shown in Table S2.

### 2.5 Luciferase assay

Osteoblastic lineage‐specific TCF/LEF reporter construct was generated using pGL3‐basic vector. Briefly, the sequence for the basal proximal promoter of rat osteocalcin gene (−199 ~ +31) that shows transcriptional activity in murine osteoblastic cells^18^ was PCR‐amplified, then cloned into pGL3‐basic vector at BglII/HindIII sites. The resulting construct was named rOC‐luc. Seven copies of TCF/LEF consensus sequence (5′‐CCTTTGATC‐3′) were amplified using two sets of PCRs with Topflash construct (Addgene) as the template to obtain two PCR fragments with different restriction sites at the ends. Then the two fragments were sequentially cloned into the above‐mentioned rOC‐luc reporter construct at the sites of KpnI/MluI and MluI/SmaI respectively. The resulting construct harboured 14 copies of TCF/LEF consensus sequence and was named 14xTCF‐rOC‐luc. The primers used are listed in Table S1.

The TCF/LEF reporter luciferase assays were carried out to assess the effect of Tax1bp3 on Wnt/β‐catenin signalling pathway. In presence of Polyfast transfection reagent (MCE), ST2 cells were transfected with 14xTCF‐rOC‐luc reporter construct (or rOC‐luc), Tax1bp3 expression construct (or pcDNA3.1 vector) and pRL‐SV40. Twenty‐four hours after transfection, the transfection medium was replaced with complete culture medium and the cells were grown in presence of 30 ng/mL recombinant Wnt3a (Peprotech) or vehicle respectively.

To study if Tax1bp3 affects BMPs/Smads signalling, a BMP‐responsive reporter construct was generated. A BMP‐responsive fragment (5′‐CTCAGACCGTTAGACGCCAGGACGGGCTGTCAGGCTGGCGCCGCCTCAGACCGTTAGACGCCAGGACGGGCTGTCAGGCTGGCGCCGCG‐3′) that contains two copies of the widely accepted BMP‐responsive element (GGCGCC)^19^, ^20^ was synthesized (Sangon Biotech), and then amplified using two sets of PCRs to obtain two PCR fragments with different restriction sites at the ends. Then the two fragments were sequentially cloned into the above‐mentioned rOC‐luc reporter construct at the sites of NheI/SmaI and KpnI/NheI respectively. The resulting construct harboured four copies of the BMP‐responsive element and was named BRE‐rOC‐luc. The primers used are listed in Table S1.

ST2 cells were transfected with BRE‐rOC‐luc luciferase reporter (or rOC‐luc), Tax1bp3 expression construct (or pcDNA3.1 vector) and pRL‐SV40 using Polyfast transfection reagent. Twenty‐four hours after transfection, the transfection medium was replaced with complete culture medium and the cells were grown in presence of 100 ng/mL recombinant BMP2 (Prospec) or vehicle, respectively. About 48 h after transfection, the cells were lysed and the luciferase activity was assayed using a dual reporter luciferase assay kit (Yeasen). The relative luciferase activity was calculated by dividing firefly luciferase activity by renilla luciferase activity.

### 2.6 Cell growth rate assay

ST2 cells were seeded in a 96‐well plate and then transfected with Tax1bp3 expression construct or vector for 4 h in presence of JetPRIME when the cells reached 70% confluence. After 24 h of incubation at 37°C, the growth rate of the cells was determined using the CCK‐8 assay kit (Dojindo Molecular Technology) following the manufacturer's instruction.

### 2.7 Alkaline phosphatase (ALP) staining and alizarin red staining

After 14 days of osteogenic treatment, the differentiated osteoblasts were briefly rinsed with phosphate buffer saline (PBS), then fixed with 4% paraformaldehyde for 15 min. The samples were then stained with NBT/BCIP staining kit to detect the presence of ALP in the cells (Beyotime Biotech). To detect mineralized nodules, the differentiated osteoblasts following 21 days of osteogenic induction were fixed with 4% paraformaldehyde and then stained with 0.1% alizarin red S solution (pH 4.2) for 5 min at room temperature.

### 2.8 Oil red O staining

After adipogenic treatment of the progenitor cells, the differentiated adipocytes were fixed with 4% paraformaldehyde for 15 min, then rinsed with deionized water. The presence of oil droplets was detected by staining the cells with 0.24% fresh oil red O solution (dissolved in 60% saturated isopropanol) for 5 min. For quantitative analysis, the retained oil red O dye in the cells was extracted with isopropanol and optical density was measured at 520 nm.

### 2.9 Western blotting

Total proteins were extracted using RIPA lysis buffer and protein concentration was measured using a BCA assay kit (Beyotime). About 30 μg proteins were separated by SDS‐PAGE and then transferred onto nitrocellulose membranes. After blocking with 5% non‐fat milk, the membranes were incubated sequentially with primary antibodies and horseradish peroxidase (HRP)‐conjugated secondary antibodies. The primary antibodies used included antibodies by Abcam: anti‐low‐density lipoprotein receptor‐related protein 6 (LRP6) (ab134146), anti‐ALP (ab108337), and anti‐osterix (ab94744); antibodies by Cell Signalling Technology: anti‐PPARγ (#2443), anti‐C/EBPα (#8178); anti‐Runx2 (#12556), anti‐phospho‐LRP6 (S1490) (#2568), anti‐non‐phospho‐β‐catenin (#8814), anti‐phospho‐GSK3β (S9) (#5558), anti‐phospho‐Smad1/5(S463/465) (#9516) and anti‐GSK3β (#12456); antibodies by Abclonal: anti‐Smad5 (A1947); antibodies by Proteintech: anti‐Smad1 (10429‐1‐AP); anti‐β‐catenin (51067‐2‐AP), anti‐osteopontin (25715‐1‐AP); anti‐fatty acid binding protein 4 (FABP4) (12802‐1‐AP), and anti‐β‐actin (66009‐1‐Ig). The bands were visualized by chemiluminescence reagent (Proteintech). The relative expression levels of the target genes were calculated by dividing the intensity of the target genes by that of β‐actin.

### 2.10 Cellular immunofluorescence

For the cellular immunofluorescence staining of β‐catenin, ST2 cells were cultured in a 24‐well plate. The cells were transfected with Tax1bp3 siRNA or control siRNA for 20 h using lipofectamine RNAi‐Max. Then the cells were fixed with 4% paraformaldehyde, treated with 1% Triton X‐100, followed by blocking with 1% bovine serum albumin. The cells were then incubated overnight at 4°C with primary antibody against β‐catenin (Proteintech). The cells were washed three times with PBS, then incubated with Alexa Fluor 488‐conjugated immunoglobulin (IgG; Proteintech) for 1 h at room temperature to detect the bound proteins. 4′,6‐diamidino‐2‐phenylindole (DAPI) was applied for counterstaining and the cells were observed under a fluorescence microscope.

### 2.11 Statistical analysis

Data are expressed as mean ± SD. For mRNA and protein quantifications, the means of the control groups were set to 1. Statistical analysis was performed with independent t test or one‐way or two‐way anova. If one‐way or two‐way anova revealed significant difference, a post hoc comparison was performed with Dunnett's test following one‐way ANOVA or Tukey's test following two‐way ANOVA. *p* < 0.05 is regarded to be significantly different.

## 3 RESULTS

### 3.1 Tax1bp3 was expressed in bone and upregulated during osteogenic and adipogenic differentiation of progenitor cells

**FIGURE 1:**
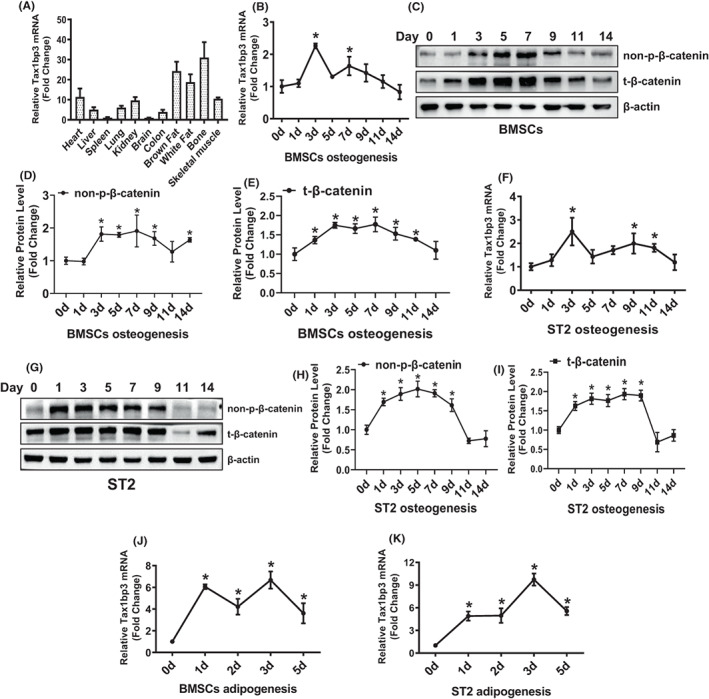
Tax1bp3 was expressed in bone and upregulated during osteogenic and adipogenic differentiation of progenitor cells. The expression levels of Tax1bp3 were detected in various tissues of mice using qRT‐PCR (A). The level of Tax1bp3 in brain was set at 1. The expression levels of Tax1bp3 and β‐catenin were assayed using qRT‐PCR and Western blotting, respectively, in primary BMSCs and ST2 cells at indicated time points after osteogenic or adipogenic induction (B–K). Values represent mean ± SD, *n* = 3. *Significant versus d 0, *p* < 0.05.

We investigated the expression levels of Tax1bp3 in various tissues of mice. The data indicated that the level of Tax1bp3 mRNA was high in bone, white and brown fat. It was moderately expressed in heart and skeletal muscle (Figure 1A). Additionally, we investigated the expression patterns of Tax1bp3 during osteogenic and adipogenic commitment of progenitor cells. The data indicated that Tax1bp3 mRNA was significantly increased at the indicated time points during osteogenic differentiation in either primary BMSCs and ST2 cells, reaching the peak level at day 3 (Figure 1B,F). As a reference, the active (non‐phosphorylated) and total forms of β‐catenin protein were increased in BMSCs and ST2 at most indicated time points of osteoblast differentiation, starting at as early as day 1 or day 3, and maintained at higher level until day 9 or even later time points (Figure 1C–E,G–I). By contrast, Tax1bp3 was also induced at the indicated time points during adipogenic differentiation in BMSCs and ST2 cells, reaching the peak at day 3 (Figure 1J,K).

### 3.2 Tax1bp3 suppressed osteogenic differentiation from progenitor cells

**FIGURE 2:**
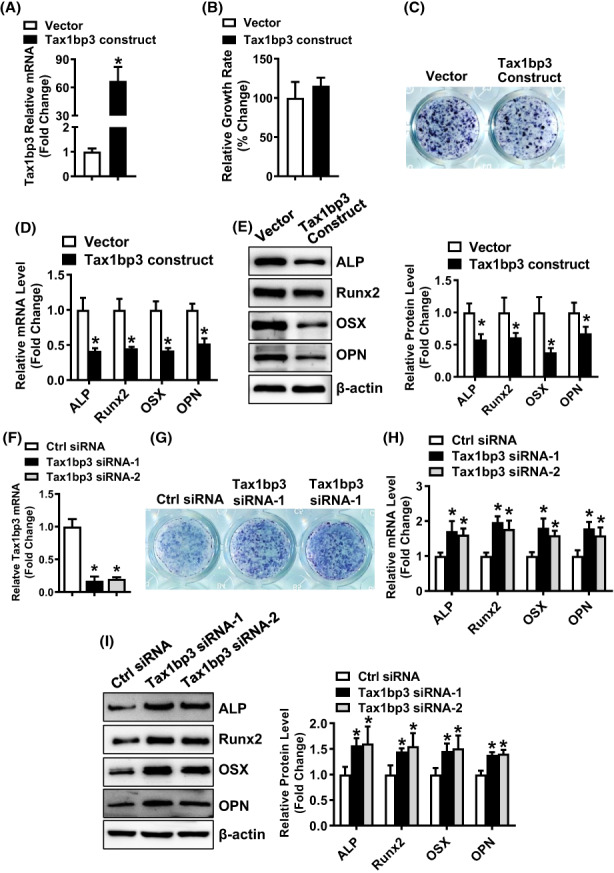
Tax1bp3 suppressed osteogenic differentiation from ST2 cells. Overexpression (A) or depletion (F) of Tax1bp3 in stromal ST2 cells after transfection of Tax1bp3 expression construct or siRNAs was verified using qRT‐PCR. The effect of Tax1bp3 on cell growth rate was examined (B). ST2 cells overexpressing or underexpressing Tax1bp3 were induced to allow osteogenic differentiation. The effects of Tax1bp3 overexpression (C–E) or depletion (G–I) on osteogenic differentiation were investigated. ALP staining was carried out in differentiated osteoblasts 14 days after osteogenic treatment (C, G). The mRNA (D, H) and protein (E, I) levels of osteogenic factors were measured by qRT‐PCR and Western blotting 72 h after osteogenic treatment. Values represent mean ± SD, *n* = 3 in (A, D, E, F, H, I), *n* = 8 in (B). *Significant versus vector or control siRNA, *p* < 0.05.

The transfection of Tax1bp3 expression construct successfully upregulated the mRNA level of Tax1bp3, as revealed by qRT‐PCR (Figure 2A). Following the overexpression of Tax1bp3, the cell growth rate of ST2 stromal cells was not affected (Figure 2B), while osteogenic differentiation of ST2 cells was attenuated in presence of osteogenic medium, as revealed by the blunted ALP staining (Figure 2C), and the decreased mRNA and protein levels of osteogenic factors such as Runx2, osterix, ALP and osteopontin in the cells overexpressing Tax1bp3 as compared to those expressing the vector (mRNAs decreased by 48 ~ 58%; proteins decreased by 33–62%) (Figure 2D,E). In contrast, the transfection of Tax1bp3 siRNAs successfully downregulated the mRNA level of Tax1bp3, as revealed by qRT‐PCR (Figure 2F). Following the depletion of Tax1bp3, osteogenic differentiation of ST2 stromal cells was stimulated in presence of osteogenic medium, as revealed by the enhanced ALP staining (Figure 2G), and the increased mRNA and protein levels of osteogenic factors such as Runx2, osterix, ALP and osteopontin in the cells transfected with Tax1bp3 siRNAs as compared to those transfected with control siRNA (mRNAs increased by 1.6 ~ 2.0‐Fold; proteins increased by 1.4 ~ 1.6‐fold, Figure 2H,I).

**FIGURE 3:**
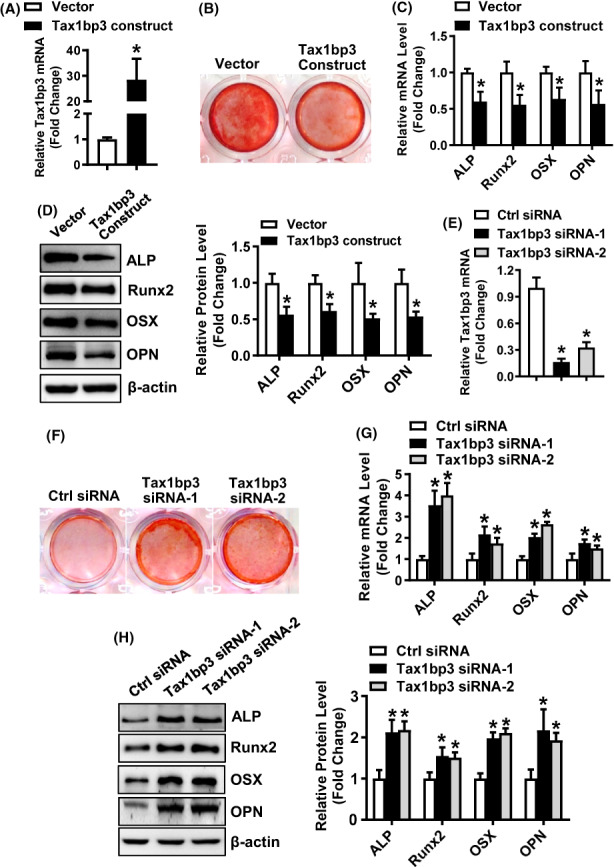
Tax1bp3 suppressed osteogenic differentiation from MC3T3‐E1 cells. Overexpression (A) or depletion (E) of Tax1bp3 in preosteoblastic MC3T3‐E1 cells after transfection of Tax1bp3 expression construct or siRNAs was verified using qRT‐PCR. MC3T3‐E1 cells overexpressing or underexpressing Tax1bp3 were induced to allow osteogenic differentiation. The effects of Tax1bp3 overexpression (B–D) or depletion (F–H) on osteogenic differentiation were investigated. Alizarin red staining were carried out in differentiated osteoblasts 21 days after osteogenic treatment (B, F). The mRNA (C, G) and protein (D, H) levels of osteogenic factors were measured by qRT‐PCR and Western blotting 72 h after osteogenic treatment. Values represent mean ± SD, *n* = 3. *Significant versus vector or control siRNA, *p* < 0.05.

Furthermore, we demonstrated that the manipulated upregulation of Tax1bp3 inhibited osteogenic differentiation of MC3T3‐E1 preosteoblastic cells and decreased the expression levels of osteogenic factors (Figure 3A–D). By contrast, the manipulated downregulation of Tax1bp3 potentiated the osteogenic differentiation of MC3T3‐E1 preosteoblastic cells and increased the expression levels of osteogenic factors (Figure 3E–H).

### 3.3 Tax1bp3 stimulated adipogenic differentiation from progenitor cells

**FIGURE 4:**
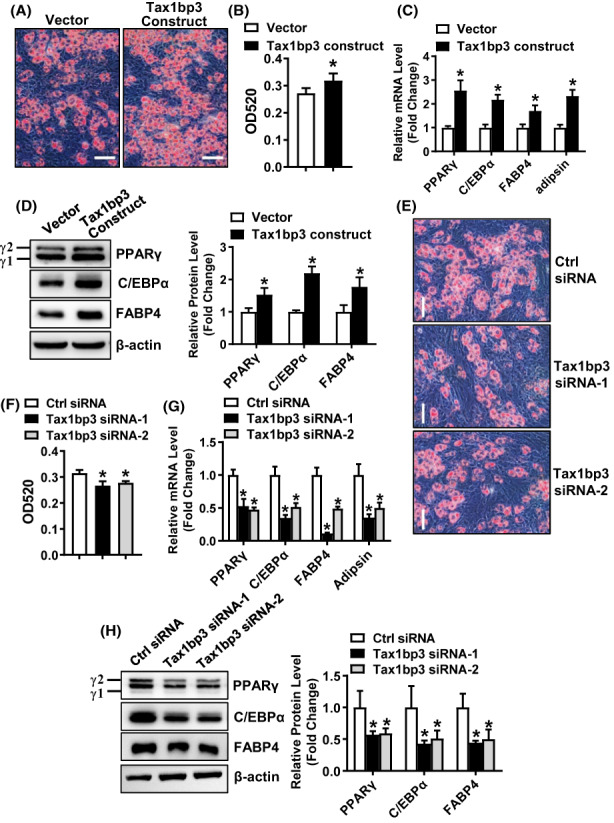
Tax1bp3 stimulated adipogenic differentiation from ST2 cells. Stromal ST2 cells were transfected with Tax1bp3 expression construct or siRNAs or their respective control, and then induced to allow adipogenic differentiation. The effects of Tax1bp3 overexpression (A–D) or depletion (E–H) on adipogenic differentiation were investigated. Oil‐red O staining was carried out in differentiated adipocytes 5 days after adipogenic treatment. Scale bar: 100 μm (A, E). The stain in the cells was extracted and OD520 was measured (B, F). The mRNA (C, G) and protein (D, H) levels of adipogenic factors were measured by qRT‐PCR and Western blotting 48 h and 72 h, respectively, after adipogenic treatment. Values represent mean ± SD, *n* = 4 in (B), *n* = 3 in (C, D, F–H). *Significant versus vector or control siRNA, *p* < 0.05.

The overexpression of Tax1bp3 stimulated adipogenic differentiation of ST2 stromal cells in presence of adipogenic medium, as revealed by the enhanced oil‐red O staining (Figure 4A,B), and the increased mRNA and protein levels of adipogenic factors such as PPARγ, C/EBPα, FABP4 and adipsin in the cells overexpressing Tax1bp3 as compared to those expressing the vector (mRNAs increased by 1.7 ~ 2.6‐Fold; proteins increased by 1.5 ~ 2.2‐fold, Figure 4C,D). In contrast, the silencing of Tax1bp3 attenuated the adipogenic differentiation of ST2 stromal cells in presence of adipogenic medium, as revealed by the blunted oil‐red O staining (Figure 4E,F), and the decreased mRNA and protein levels of adipogenic factors in the cells transfected with Tax1bp3 siRNAs as compared to those transfected with control siRNA (mRNAs decreased by 47 ~ 89%; proteins decreased by 41 ~ 57%, Figure 4G,H).

**FIGURE 5:**
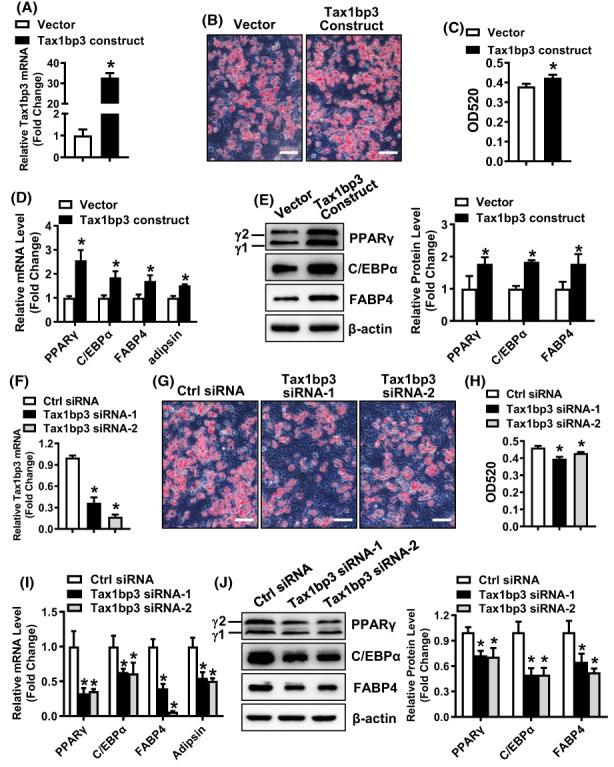
Tax1bp3 stimulated adipogenic differentiation from C3H10T1/2 cells. Overexpression (A) or depletion (F) of Tax1bp3 in mesenchymal C3H10T1/2 cells after transfection of Tax1bp3 expression construct or siRNAs was verified using qRT‐PCR. C3H10T1/2 cells were transfected with Tax1bp3 expression construct or siRNAs or their respective control, and then induced to allow adipogenic differentiation. The effects of Tax1bp3 overexpression (B–E) or depletion (G–J) on adipogenic differentiation were investigated. Oil‐red O staining was carried out in differentiated adipocytes 5 days after adipogenic treatment. Scale bar: 100 μm (B, G). The stain in the cells was extracted and OD520 was measured (C, H). The mRNA (D, I) and protein (E, J) levels of adipogenic factors were measured by qRT‐PCR and Western blotting 48 h and 72 h, respectively, after adipogenic treatment. Values represent mean ± SD, *n* = 3. *Significant versus vector or control siRNA, *p* < 0.05.

Furthermore, we demonstrated that the manipulated upregulation of Tax1bp3 stimulated adipogenic differentiation of C3H10T1/2 mesenchymal cells and increased the expression levels of adipogenic factors (Figure 5A–E). By contrast, the manipulated downregulation of Tax1bp3 suppressed adipogenic differentiation of C3H10T1/2 cells and decreased the expression levels of adipogenic factors (Figure 5F–J).

### 3.4 Overexpression of Tax1bp3 in preosteoblastic cells of mice downregulated osteogenesis and stimulated adipogenesis

**FIGURE 6:**
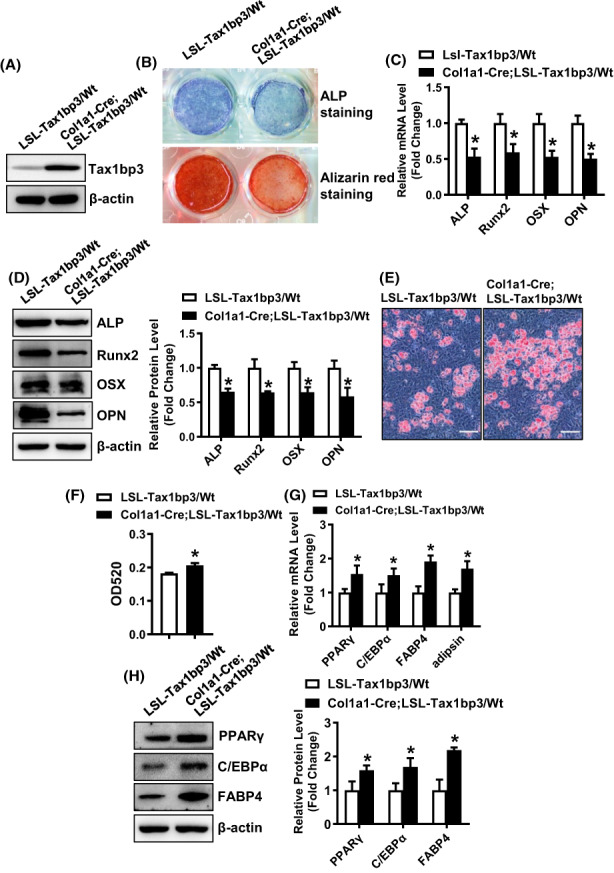
Overexpression of Tax1bp3 in preosteoblastic cells of mice downregulated osteogenesis and stimulated adipogenesis. The primary calvarial cells were isolated from Col1a1‐Cre; LSL‐Tax1bp3/Wt mice and the control LSL‐Tax1bp3/Wt mice. The overexpression of Tax1bp3 in osteoblastic cells was verified using Western blotting (A). The cells were induced to allow osteogenic or adipogenic differentiation. ALP staining and alizarin red staining were carried out in differentiated osteoblasts 14 and 21 days, respectively, after osteogenic treatment (B). The mRNA (C) and protein (D) levels of osteogenic factors were measured by qRT‐PCR and Western blotting, respectively, 72 h after osteogenic treatment. Oil‐red O staining was carried out in differentiated adipocytes 5 days after adipogenic treatment. Scale bar: 100 μm (E). The quantity of the stain in the cells was measured at OD520 (F). The mRNA (G) and protein (H) levels of adipogenic factors were measured by qRT‐PCR and Western blotting 48 h and 72 h, respectively, after adipogenic treatment. Values represent mean ± SD, *n* = 3. *Significant versus LSL‐Tax1bp3/Wt, *p* < 0.05.

The strategy for generating LSL‐Tax1bp3 mice was shown in Figure S1A. To investigate the in vivo function of Tax1bp3 in the regulation of osteogenesis, we generated a conditional knock‐in strain with overexpression of Tax1bp3 specifically in preosteoblastic cells through crossing LSL‐Tax1bp3 mice with Col1a1‐Cre mice. The genotypes of the mice were confirmed using genotyping PCR (Figure S1B). The primary calvarial cells were isolated from Col1a1‐Cre; LSL‐Tax1bp3/Wt mice and the control LSL‐Tax1bp3/Wt mice. The overexpression of Tax1bp3 in calvarial osteoblastic cells was verified using Western blotting (Figure 6A). Upon osteogenic treatment, the cells overexpressing Tax1bp3 showed blunted ALP staining and alizarin red staining and decreased expression levels of osteogenic factors (Figure 6B–D). Upon adipogenic treatment, the cells overexpressing Tax1bp3 exhibited enhanced oil‐red O staining and increased expression levels of adipogenic factors (Figure 6E–H).

### 3.5 Tax1bp3 inactivated Wnt/β‐catenin and BMPs/Smads signalling

**FIGURE 7:**
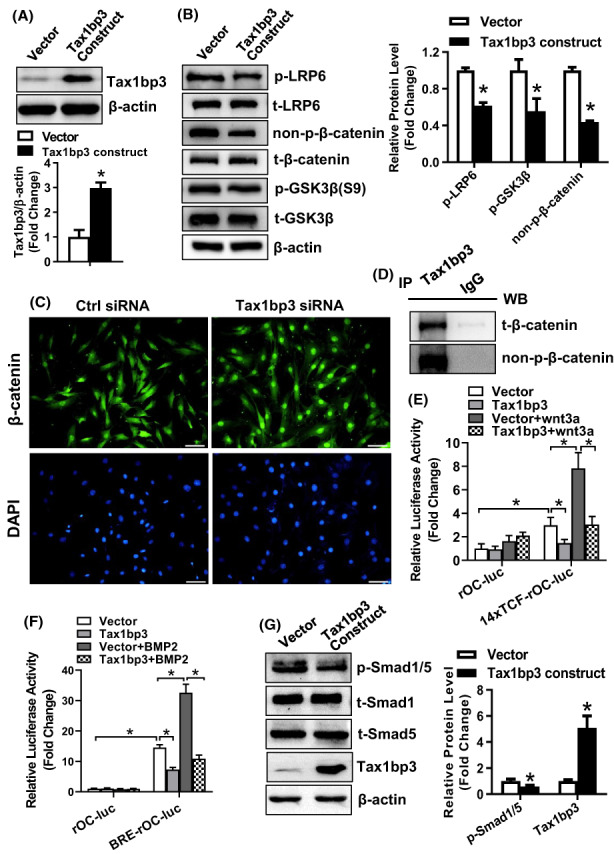
Tax1bp3 inactivated Wnt/β‐catenin and BMPs/Smads signalling. The protein levels of Tax1bp3 (A) and the major components of canonical Wnt signalling (B) were measured using Western blotting in undifferentiated ST2 72 h after overexpression of Tax1bp3. The nuclear translocation of β‐catenin was detected in ST2 cells transfected with Tax1bp3 siRNA. Image scale: 50 μm (C). Co‐IP was done to detect the interaction between Tax1bp3 and β‐catenin (D). The relative luciferase activity of 14xTCF‐rOC‐luc reporter (E) or BRE‐rOC‐luc reporter (F) in absence or presence of Wnt3a or BMP2 was assayed in ST2 cells 48 h after transfection. The protein levels of the Smad1/5 were measured using Western blotting in undifferentiated ST2 72 h after overexpression of Tax1bp3 (G). Values represent mean ± SD, *n* = 3 in (A, B, G), *n* = 5 in (E, F). *p < 0.05.

To uncover the mechanism underlying the control of stromal progenitor cell differentiation, the status of Wnt/β‐catenin signalling was checked in ST2 cells following overexpression and/or silencing of Tax1bp3. It was shown by Western blotting analysis that Tax1bp3 overexpression significantly reduced the protein levels of phospho‐LRP6(S1490), phospho‐GSK3β(S9) and active non‐phospho‐β‐catenin (Figure 7A,B). Consistently, it was shown by immunofluorescence staining that the depletion of Tax1bp3 stimulated the translocation of β‐catenin to the nuclei from the cytoplasm (Figure 7C). Furthermore, co‐IP experiment revealed that Tax1bp3 binds with β‐catenin (Figure 7D). TCF/LEF reporter luciferase assay revealed that Wnt3a increased the relative luciferase activity of 14xTCF‐rOC‐luc reporter in ST2 cells by 2.6‐fold, and Tax1bp3 overexpression downregulated the relative luciferase activity of 14xTCF‐rOC‐luc reporter by 51% in absence of Wnt3a stimulation, and by 61% in presence of Wnt3a stimulation (Figure 7E). In contrast, BMP2 increased the relative luciferase activity of the BMPs/Smads reporter, that is, BRE‐rOC‐luc, by 2.2‐fold, and Tax1bp3 overexpression downregulated the relative luciferase activity of the BRE‐rOC‐luc reporter by 50% in absence of BMP2 stimulation, and by 67% in presence of BMP2 stimulation (Figure 7F). Moreover, Western blotting data showed that Tax1bp3 overexpression significantly reduced the protein levels of phosphorylated Smad1/5(S463/465) (Figure 7G).

## 4 DISCUSSION

Tax1bp3 was initially identified as a player in the proliferation and growth of colorectal cancer cells.^21^ Efforts have been made to clarify the regulatory role of Tax1bp3 in tumorigenesis and metastasis of cancers.^12^, ^13^, ^14^, ^22^, ^23^ Recently there is an emerging interest in the function of Tax1bp3 in the cellular processes other than tumorigenesis. Reinstein et al. has recently reported a novel autosomal recessive syndrome manifested dilated cardiomyopathy, agenesis of the corpus callosum and septo‐optic dysplasia caused by a homozygous missense mutation in Tax1bp3.^9^ These findings appear to be consistent to Besser et al.'s report that the knockdown of Tax1bp3 resulted in elongation defects, enlarged pericardium and enlarged head structures in zebrafish embryos.^10^ Collectively, these evidences highlight the important role of Tax1bp3 for heart and brain development. However, it is not known if and how Tax1bp3 acts in the cell fate decision of bone marrow mesenchymal progenitor cells.

In the current study, we have explored the expression profiling of Tax1bp3 in mice and its function in the differentiation of mesenchymal progenitor cells. It was shown that Tax1bp3 was abundant in bone and was upregulated during both osteoblast differentiation and adipocyte formation. The gain‐of‐function and loss‐of‐function experiments using stromal ST2 cells and MC3T3‐E1 cells revealed that Tax1bp3 suppressed osteoblast differentiation. By contrast, the functional studies using ST2 and C3H10T1/2 unravelled the stimulatory role of Tax1bp3 in adipocyte differentiation. Of more importance, we also performed the functional experiment using the calvarial cells isolated from the conditional Tax1bp3 knock‐in mice, which further demonstrated the function of Tax1bp3 as a suppressor of osteoblast differentiation and promoter of adipocyte formation.

Wnt signalling is vital for the development and remodelling of bone.^24^, ^25^ It regulates the differentiation of the major effector cells of bone, that is, osteoblasts and osteoclasts.^26^, ^27^, ^28^ The deregulation of Wnt/β‐catenin pathway leads to the impaired differentiation of bone cells and impaired bone mass accrual.^25^, ^28^ It was documented in Kanamori et al.'s study that Tax1bp3 was able to bind with β‐catenin and the interaction is mediated by the PDZ domain of Tax1bp3 and requires primarily the last four amino acids of β‐catenin. Further investigation revealed that Tax1bp3 suppressed the transcriptional activity of β‐catenin.^21^ Consistently, using TCF/LEF reporter, our study also indicated the inhibitory effect of Tax1bp3 on the transcriptional activity of Wnt/β‐catenin signal. We further checked the relationship between Tax1bp3 and canonical Wnt signalling and found that Tax1bp3 was able to inactivate Wnt/β‐catenin pathway and block the nuclear translocation of β‐catenin from the cytoplasm, suggesting the involvement of Wnt/β‐catenin pathway in the altered osteogenic and adipogenic differentiation of mesenchymal progenitor cells induced by Tax1bp3. The co‐IP experiment revealed the physical binding of Tax1bp3 and β‐catenin. This finding is consistent with Kanamori et al.'s study and suggests that the binding of Tax1bp3 to β‐catenin may interfere the nuclear translocation of the latter, thus blocking the activation of canonical Wnt signalling. However, this probably is not the unique mechanism for the inactivation of Wnt/β‐catenin pathway, because Tax1bp3 overexpression led to the downregulation of phospho‐LRP6(S1490) and phospho‐GSK3β(S9), suggesting that there is also a mechanism upstream of β‐catenin that is responsible for the inactivation of canonical Wnt signalling by Tax1bp3. Since Runx2 and osterix are the direct downstream transcriptional targets of Wnt/β‐catenin,^29^, ^30^, ^31^ Tax1bp3 regulated Runx2 and osterix expression at least partially through its effect on Wnt/β‐catenin signalling.

Of note, Tax1bp3 downregulated the phosphorylated levels of BMP responsive Smads. Additionally, using a luciferase reporter containing multiple copies of previously identified BMP responsive enhancer sequence,^19^, ^20^ we found that Tax1bp3 was able to downregulate the activity of the reporter. The data suggest that inhibition of BMPs/Smads pathway may also be a downstream mediator of Tax1bp3 function. However, unlike the reciprocal role of many other regulators in osteogenesis and adipogenesis, several BMP subfamily members including BMP‐2, BMP‐4 and BMP‐7 induce osteogenic differentiation,^32^, ^33^ as well as adipogenic commitment and/or differentiation from mesenchymal stem cells.^34^, ^35^, ^36^ This contradicts the reciprocal role of Tax1bp3, suggesting that inhibition of BMPs/Smads signalling may be involved in the function of Tax1bp3 in osteoblast differentiation but not in adipocyte differentiation.

In summary, we have for the first time identified that Tax1bp3 is a reciprocal regulator of osteogenic and adipogenic differentiation from mesenchymal progenitor cells. The underlying mechanism for its reciprocal function may involve its interaction with Wnt/β‐catenin signalling pathway. Taken as a whole, our study may suggest a potential target for the treatment of metabolic bone disorders such as osteoporosis. A weakness of the current study is that the bone phenotypes of the conditional knock‐in mice are not herein reported, which will be focused on in our future investigations. Moreover, the involvement of Wnt/β‐catenin signalling in the in vivo role of Tax1bp3 is to be further explored.

## AUTHOR CONTRIBUTIONS

**Yi Wang:** Conceptualization (equal); data curation (equal); investigation (lead); methodology (equal). **Ying Gan:** Conceptualization (equal); data curation (equal); investigation (supporting); methodology (equal). **Yuan Dong:** Conceptualization (equal); data curation (equal); investigation (supporting); methodology (equal). **Jie Zhou:** Funding acquisition (equal); methodology (equal). **Endong Zhu:** Funding acquisition (equal); methodology (equal). **Hairui Yuan:** Conceptualization (equal); supervision (equal). **Xiaoxia Li:** Conceptualization (equal); supervision (equal). **Baoli Wang:** Conceptualization (equal), funding acquisition (equal), project administration (lead), writing‐review & editing (lead).

## CONFLICT OF INTEREST

The authors declare no conflict of interests.

## Supporting information


Appendix S1.

## Data Availability

The data that support the findings of this study are available from the corresponding author upon reasonable request.
