# Supplementary material for: Tax1 binding protein 3 regulates osteogenic and adipogenic differentiation through inactivating Wnt/β‐catenin signalling
Source: J Cell Mol Med. 2023 Mar 9;27(7):950–61. doi: 10.1111/jcmm.17702 (PMC10064035; doi:10.1111/jcmm.17702)
Supplement: Supplementary file 1 — Appendix S1. [file JCMM-27-950-s001.pdf]

Figure S1

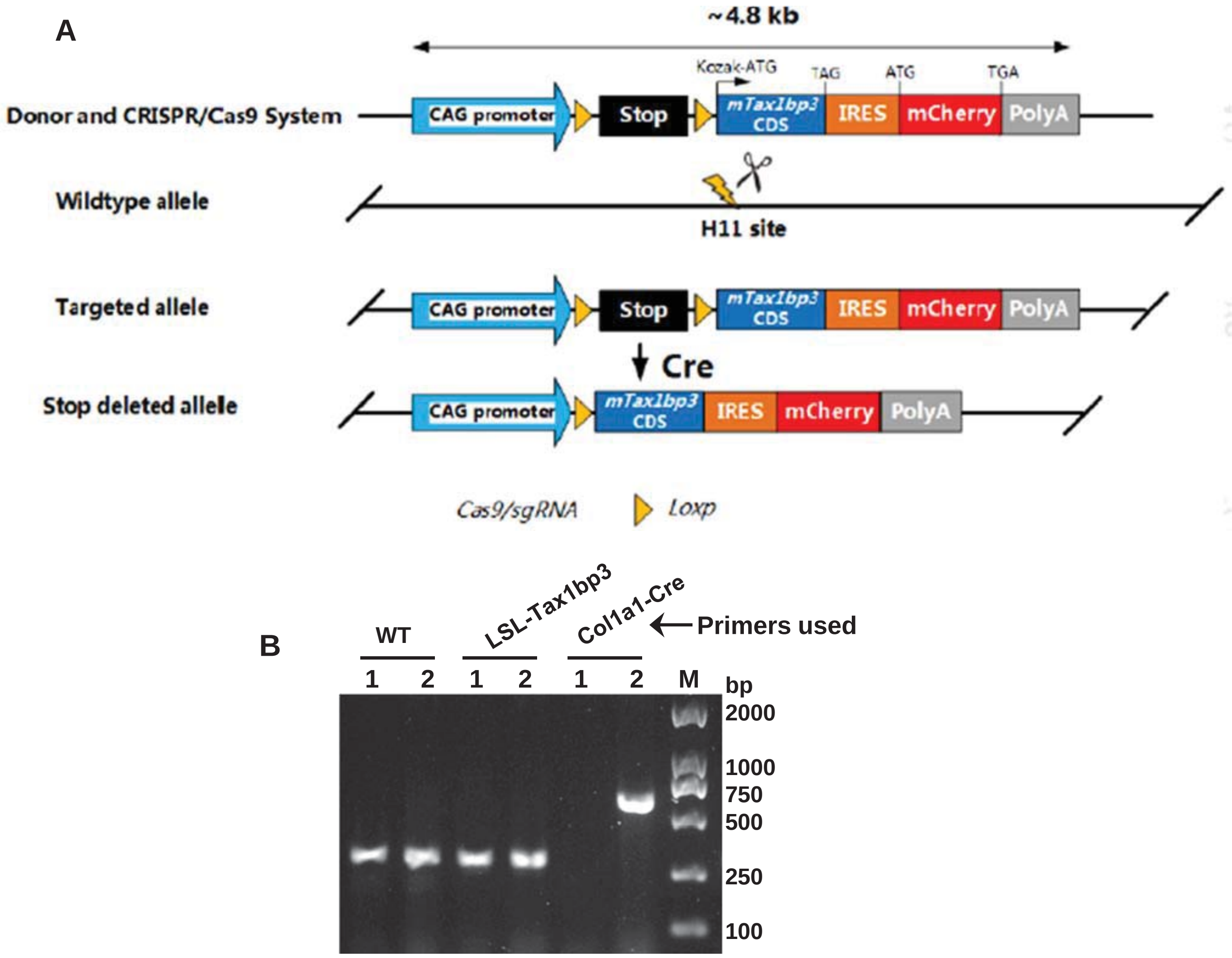

Fig. S1. Generation of conditional Tax1bp3 knock-in mice. The strategy for making LSL-Tax1bp3 mice is shown (A). The genotypes of the mutant mice were confirmed by genotyping PCR (B). Lables 1: LSL-Tax1bp3/Wt; 2: Col1a1-Cre; LSL-Tax1bp3/Wt.

Table S1. Primers used for RT-PCR, cloning and genotyping

Genes	Forward primer sequences:	Reverse primer sequences
PPARγ	CTTGACAGGAAAGACAACGG	GCTTCTACGGATCGAAACTG
C/EBPα	CTGATTCTTGCCAAACTGAG	GAGGAAGCTAAGACCCACTAC
FABP4	AAATCACCGCAGACGACAGG	GGCTCATGCCCTTTCATAAAC
adipsin	TGATGTGTGCAGAGAGCAAC	CGTAACCACACCTTCGACTG
Runx2	TCCTGTAGATCCGAGCACCA	CTGCTGCTGTTGTTGCTGTT
ALP	CCAGAAAGACACCTTGACTGTGG	TCTTGTCCGTGTCGCTCACCAT
Osterix	GGCTTTTCTGCGGCAAGAGGTT	CGCTGATGTTTGCTCAAGTGGTC
OPN	GCTTGGCTTATGGACTGAGGTC	CCTTAGACTCACCGCTCTTCATG
β-actin	AAGACCTCTATGCCAACACAG	GGAGGAGCAATGATCTTGATC
Tax1bp3 full length	TTGGTACCGAGCTCGGATCCGCCACCATGTCCTACACCCCGGGCCA	GCTGGATATCTGCAGAATTCCTAAGACAGCATGGACTGCT
rOC promoter	GCGCCCGCGTGCTAGATCTACCCGGCAGCCTCTGATTGTG	AGTACCGGAATGCCAAGCTTTGTCTGCTAGGTCTGCACCG
14xTCF PCR1	TTTCTCTATCGATAGGTACCAGCTCTTACGCGAGATCA	AGCCCGGGCTAGCACGCGTAGCACGCGGGCGCGCCCCT
14xTCF PCR2	CGCGCCCGCGTGCTACGCGTAGCTCTTACGCGAGATCA	TCGCAGATCTCGAGCCCGGGAGCACGCGGGCGCGCCCCT
BRE PCR1	AGCTCTTACGCGTGCTAGCCTCGATCTCAGACCGTTAGACGCCAG	GGGTAGATCTCGAGCCCGGGCGCGGCGCCAGCCTGACAG
BRE PCR2	TTTCTCTATCGATAGGTACCCTCGATCTCAGACCGTTAGACGCC	GGTCTGAGATCGAGGCTAGCCGCGGCGCCAGCCTGACAGC
WT genotyping	GGGCAGTCTGGTACTTCCAAGCT	ATATCCCCTTGTTCCCTTTCTGC
LSL-Tax1bp3 genotyping	TGATCCGGAACCATAACTTC	AGCCCAGCAATTTCAGCAGG
Col1a1-Cre genotyping	CCCAGCTCTCCATCAAGATG	GTGAAACAGCATTGCTGTCACTT

Table S2. siRNA sequences used for gene silencing

Genes	Sense sequences	Antisense sequences
Tax1bp3 siRNA-1	GGGAGGUCCUGCUGAAAUUTT	AAUUUCAGCAGGACCUCCCTT
Tax1bp3 siRNA-2	GGCUGCAGAUUGGAGACAATT	UUGUCUCCAAUCUGCAGCCTT
